# Macronutrients in Human Milk Exposed to Antidepressant and Anti-Inflammatory Medications

**DOI:** 10.1001/jamanetworkopen.2024.53332

**Published:** 2025-01-07

**Authors:** Essi Whaites Heinonen, Kerri Bertrand, Christina Chambers

**Affiliations:** 1 Department of Clinical Science, Intervention and Technology, Division of Pediatrics, Karolinska Institutet, Stockholm, Sweden; 2 Department of Pediatrics, Center for Better Beginnings, University of California, San Diego, La Jolla

## Abstract

**Importance:**

The association between maternal medications and the macronutrient composition of human milk has not been studied.

**Objective:**

To compare macronutrient levels in milk samples from mothers treated with long-term medications with samples from untreated healthy and disease-matched control mothers (DMCs).

**Design, Setting, and Participants:**

A cross-sectional study using samples collected between October 2014 and January 2024 from breastfeeding mothers in the US and Canada invited to participate to the Mommy’s Milk Human Milk Research Biorepository at the University of California, San Diego. Of 3974 samples from unique individuals in the biorepository, 310 were from mothers treated with 1 of 4 categories of medications, 151 from DMCs with the same underlying disorders, and 73 from healthy untreated mothers, frequency matched on infant age and sex. Of these, 150 were excluded because they had more than 1 medication exposure or were outliers. Data were analyzed from March to June 2024.

**Exposures:**

Continuous treatment with selective serotonin reuptake inhibitors (SSRIs), monoclonal antibodies (MABs), systemic steroids, and other anti-inflammatory drugs (ADs) in the 14 days before milk sample collection.

**Main Outcomes and Measures:**

Levels of protein, fat, carbohydrate, and total energy were measured with SpectraStar 2400 near infrared analyzer and compared across groups with analysis of covariance adjusted for infant and maternal age, parity, maternal body mass index, infant sex, exclusive breastfeeding, feeding frequency, collection time, maternal cannabis use, and occupation.

**Results:**

A total of 384 samples were collected; 194 infants (50.5%) were female; the mean (SD) age of the maternal cohort was 33.5 (4.4) years, and infant age at collection was 6.6 (5.4) months. Mean (SD) protein levels were 15% to 21% lower in samples from exposed mothers (0.92 [0.56] g/100 mL for 63 SSRIs, 0.85 [0.51] g/100 mL for 63 MABs, 0.88 [0.37] g/100 mL for 33 steroids, and 0.85 [0.54] g/100 mL for 20 other ADs) compared with 64 samples from healthy mothers (1.08 [0.50] g/100 mL). Adjusted differences were significant for SSRIs and steroids (*F*_1, 91_ = 4.32; *P* = .04 and *F*_1,59_ = 5.00, *P* = 0.03, respectively). Mean (SD) fat and energy were 10% to 22% lower in samples from mothers with other ADs (3.40 [1.21] g/100 mL for fat and 69.56 [15.35] kcal/100 mL for energy) than from healthy (3.85 [1.66] g/100 mL for fat and 77.16 [22.08] kcal/100 mL for energy) and DMC (4.38 [1.90] g/100 mL for fat and 80.60 [24.70] kcal/100 mL for energy) mothers. Adjusted differences were only significant for fat compared with DMC (*F*_1,88_ = 6.22; *P* = .01).

**Conclusions and Relevance:**

In this cross-sectional study, some maternal medications were associated with lower levels of protein and fat in milk, which could impose health risks for breastfed infants. Other factors that could influence macronutrient levels need to be clarified before the clinical implications of these findings can be confirmed.

## Key Points

**Question:**

Is maternal treatment with long-term medications associated with changes in the macronutrient composition of human milk?

**Findings:**

In this cross-sectional study including milk samples from 63 mothers treated with antidepressants, 106 with long-term anti-inflammatory medications, 141 untreated mothers with the same underlying disorders, and 64 healthy matched mothers, lower levels of protein and fat, but not carbohydrates, were associated with some of the medications.

**Meaning:**

These findings suggest maternal medications may alter the macronutrient composition of human milk.

## Teaser

This cross-sectional study evaluates macronutrient levels in milk samples from mothers treated with long-term medications and compares them with samples from untreated healthy and disease-matched mothers.

## Introduction

Breastfeeding is known to have several health benefits for both mother and child, and is the recommended sole source of nutrition for the first 6 months of life.^1^ The nutritional components of human milk are carbohydrates mainly consisting of lactose (8 g/100 mL), fat (3.5-4 g/100 mL), and protein (1 g/100 mL), with a total energy content around 66 kcal/100 mL (19 kcal/oz).^2,3,4,5^ There is natural variation in macronutrient levels in human milk based on time of day, times fed per day, size of the milk portion, infant age, maternal age, maternal diet, maternal body mass index (BMI), and infant weight percentile.^3,4,6,7,8,9,10^ The method of analysis also affects the accuracy of the results.^11,12^

Generally, the macronutrient composition of human milk is well tailored to match the needs of the breastfed infant, and the clinical implications of the varying macronutrient levels for the breastfed infant are mainly studied in the context of donor milk banks.^2,4,10,11^ The carbohydrate levels in human milk seem positively correlated with infant weight and protein with infant length. The levels of fat are the main determinant of the total energy of human milk and are known to aid in the development of the central nervous system of the breastfed infant.^2,4,10,13^

More than 70% of women who provide human milk to their infants take some form of medication.^14^ Whether external exposures could affect the composition of human milk is only studied in a few single studies, with cannabis suggested to affect the levels of protein and carbohydrates by some, but not confirmed by others.^15,16^

However, physiologic and biochemical phenomena that influence the composition of plasma may also affect the composition of milk.^17^ Selective serotonin reuptake inhibitors (SSRIs) are suggested to increase the risk of hypoglycemia. Similarly, treatment with systemic steroids is known to cause hyperglycemia, and some monoclonal antibodies (MABs) are suggested to cause hyperglycemia. For these reasons, there might be at least a theoretical risk of altered macronutrient levels in human milk produced by mothers treated with these medications.^18,19,20,21^ However, the concentrations of lactose are known to be fairly constant in mature milk (ie, after 21 days post partum).^10^

To our knowledge, the association of prescription medications with human milk composition has not been studied previously. We aimed to compare macronutrient levels in human milk from mothers exposed to long-term antidepressant and anti-inflammatory medications with levels in milk from disease-matched and healthy mothers.

## Methods

### Study Setting and Ethics

Exposure data and milk samples for this cross-sectional study were collected between October 10, 2014 and January 23, 2024, from mothers in the US and Canada through The University of California, San Diego Human Milk Biorepository (HMB).^22,23^ This study was approved by the University of California, San Diego institutional review board. All study participants provided written informed consent. All procedures followed were in accordance with the ethical standards of the responsible committee on human experimentation (institutional and national) and with the Helsinki Declaration of 1975, as revised in 2000. This report follows the Strengthening the Reporting of Observational Studies in Epidemiology (STROBE) reporting guideline for cross-sectional studies.^24^

### Participants

**Figure 1.**
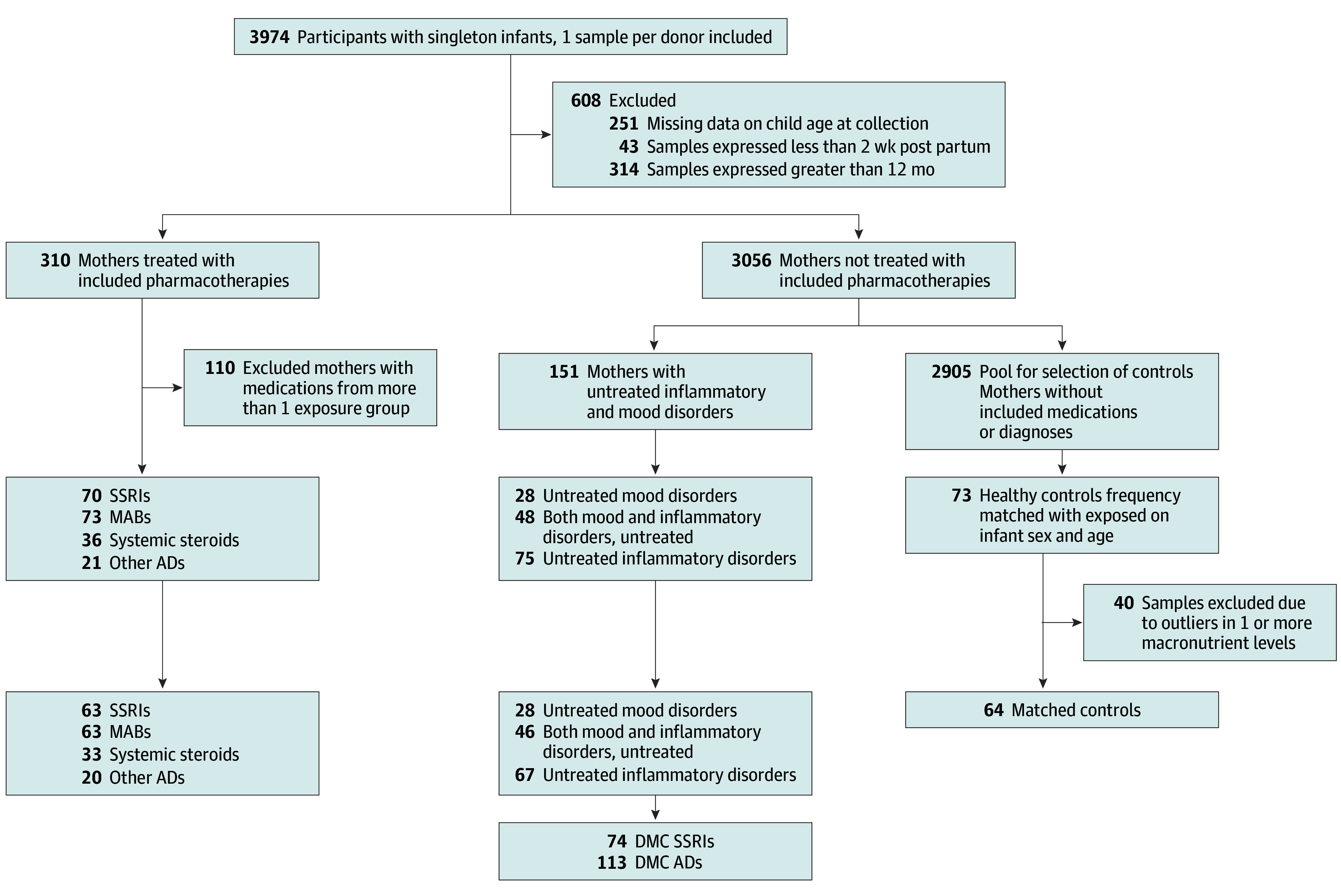
Flow Chart of the Study Cohort ADs indicates anti-inflammatory drugs; DMC, disease matched control group; MABs, monoclonal antibodies; SSRIs, selective serotonin reuptake inhibitors.

Data and samples provided by previously consented participants were obtained from the HMB database. Mothers of singleton infants were considered for inclusion, with the first sample for each eligible mother selected. Samples with missing data on child age at sample collection and samples collected before 2 weeks and after 12 months post partum were excluded (Figure 1). Of the 3366 milk samples that matched the inclusion criteria, 310 were from mothers treated long-term with any of the selected medications and 3056 not treated. The selected long-term medication groups were classified into 4 mutually exclusive groups: SSRIs, MABs, systemic steroids, and other anti-inflammatory drugs (ADs). Out of the 310 medicated mothers, 110 were excluded due to treatment with medications from more than 1 exposure group.

Information on maternal medical disorders was collected by maternal interview near the time of sample collection. Participants were also screened for current mood disorders by completing an online version of the Edinburgh Postnatal Depression Scale (EPDS) near the time of sample collection.^25^ Based on these data, 123 of the untreated mothers were identified as being diagnosed with inflammatory disorders (including rheumatic and inflammatory bowel disorders [IBD]), and 76 with mood disorders, including mothers with psychiatric diagnoses and mothers who scored 10 or more or answered yes to having suicidal ideation on the EPDS. These mothers were defined as disease-matched controls (DMCs), and the 48 mothers with both inflammatory and mood disorders were included in both the DMC comparison groups for the SSRIs and for the ADs (Figure 1). The remaining 2905 mothers without exposure to the selected medications or the underlying disorders comprised a pool for selection of a healthy control group. The healthy control group members were frequency-matched on infant age and sex to the largest group of mothers treated with MABs, and for purposes of efficacy, the same healthy control group was used for the other exposure groups.

### Exposures

Data on exposure to prescription medications were reported by the mothers in telephone interviews at the time of sample collection. Mothers treated with the selected medications continuously in the 14 days before sample collection were considered exposed. For MABs administered every 4 to 8 weeks, the mothers were considered exposed if they were continuously treated with the MAB and were in steady state at collection of milk sample, even if the MAB was not administered in the last 14 days. The exposure groups were defined as: (1) SSRIs; (2) MABs; (3) corticosteroids administered systemically (ie, orally or intravenously), and (4) other ADs, including conventional disease-modifying antirheumatic agents and other anti-inflammatory agents used in treatment of IBD, such as mesalazine.

### Main Outcome Measures

The main outcome measures were the levels of carbohydrates, fat, and protein, measured in g/100 mL, and total energy, measured in kcal/100 mL, in human milk. For the analysis, frozen milk samples were prewarmed to 40 °C in a digital water bath and homogenized using a sonicator to prevent milk fat from separating out.^26^ After sonification, 1 mL of milk was drawn up with an electronic pipet and expelled onto a gold transflectance chamber that was magnetically sealed carefully so as not to trap air bubbles.^26^ The chamber was loaded into the SpectraStar 2400 near infrared machine (Unity Scientific) and spectrophotometric analysis was performed across the entire spectrum between 1200 to 2400 nm. Individual full spectra (1200 to 2400 nm) for each sample were compared with the reference chemistry values for each macronutrient (fat, protein, and carbohydrate) and then analyzed by Uscan software version 3.0.2.4 (Unity Scientific) that uses partial squares method to determine the best correlation.^26^

### Covariates

Data on background characteristics and covariates were collected through maternal telephone interviews and are presented in Table 1. Self-reported maternal race and ethnicity were categorized as Asian, non-Hispanic Black, Hispanic, non-Hispanic White, and Other, which included American Indian and Native Hawaiian/Pacific Islander. Race was assessed in this study because of differences in human milk composition between different race and ethnicity groups presented by some studies.^27^ Covariates included in the adjusted analyses were infant age at sample collection in months (continuous), maternal age in years (continuous), parity (binary or primipara vs multipara), maternal BMI, calculated as weight in kilograms divided by height in meters squared (continuous), infant sex (binary), exclusive breastfeeding defined as breastmilk only vs breastmilk together with formula and/or solid foods (binary), times fed per day (continuous), collection time of milk sample (continuous), maternal use of cannabis in the previous 14 days (binary, yes or no) and maternal occupation (categorical, full-time, part-time, or unemployed).

**Table 1.** Background Characteristics of the Study Population

Characteristic	Participants, No. (%)^a^							*P* value between groups^c^
	Total cohort (n = 384)	SSRIs (n = 63)	MABs (n = 63)	Steroids (n = 33)	Other ADs (n = 20)	Disease-matched control group (n = 141)^b^	Healthy control group (n = 64)	
Race and ethnicity								
Asian	27 (7.0)	2 (3.2)	2 (3.2)	1 (3.0)	0	15 (10.6)	7 (10.9)	.12
Black non-Hispanic	6 (1.6)	0	1 (1.6)	0	0	3 (2.1)	2 (3.1)	
Hispanic	48 (12.5)	7 (11.0)	6 (9.5)	6 (18.2)	1 (5.0)	18 (12.8)	10 (15.6)	
White non-Hispanic	291 (75.8)	52 (82.5)	52 (82.5)	26 (78.8)	16 (80.0)	101 (71.6)	44 (68.8)	
Other^d^	11 (2.9)	2 (3.2)	1 (1.6)	0	3 (15.0)	4 (2.8)	1 (1.6)	
Primipara	182 (47.4)	30 (47.5)	39 (61.9)	15 (45.5)	8 (40.0)	66 (46.8)	24 (37.5)	.14
Infant sex								
Female	194 (50.5)	29 (46.0)	25 (39.7)	20 (60.6)	13 (65.0)	72 (51.1)	35 (54.7)	.22
Male	190 (49.5)	34 (54.0)	38 (60.3)	13 (39.4)	7 (35.0)	69 (48.9)	29 (45.3)	
Exclusive breast milk	143 (37.2)	20 (31.7)	31 (49.2)	12 (36.4)	8 (40.0)	51 (36.4)	21 (33.3)	.39
Sample collection time, mean (SD), h	11.8 (4.0)	12.4 (3.9)	11.9 (4.8)	11.8 (3.6)	11.2 (5.1)	11.7 (3.7)	11.8 (3.9)	.86
Data available	339 (88.3)	59 (93.7)	52 (82.5)	26 (78.8)	17 (85.0)	128 (90.8)	57 (89.1)	NA
Mood disorders^e^	167 (384)	58 (92.1)	18 (28.6)	12 (36.4)	5 (25.0)	74 (52.5)	0	<.001
Inflammatory disorders	246 (64.1)	43 (68.3)	58 (92.1)	15 (45.5)	18 (90.0)	112 (79.4)	0	<.001
Tobacco use	12 (3.1)	1 (1.6)	0	1 (3.0)	0	6 (4.3)	4 (6.3)	.32
Cannabis use	59 (15.4)	14 (22.2)	2 (3.2)	0	2 (10.0)	24 (17.0)	17 (26.6)	<.001
Maternal occupation								
Data available	346 (90.1)	59 (93.7)	59 (93.7)	28 (84.8)	18 (90.0)	123 (87.2)	59 (92.2)	NA
Full time	189 (49.2)	27 (45.8)	38 (64.4)	18 (64.3)	7 (38.9)	69 (56.1)	30 (50.8)	.41
Part time	78 (20.3)	15 (25.4)	10 (16.9)	4 (14.3)	6 (33.3)	23 (18.7)	20 (33.9)	
Unemployed	79 (20.6)	17 (28.8)	11 (18.6)	6 (21.4)	5 (27.8)	31 (25.2)	9 (15.3)	
Household income/y, US $								
Data available	336 (87.5)	55 (87.3)	60 (95.2)	32 (97.0)	19 (95.0)	115 (81.6)	55 (85.9)	.92
≤50 000	10 (3.0)	1 (1.8)	1 (1.7)	0	0	5 (4.3)	3 (5.5)	
50 000-99 999	161 (47.9)	28 (50.9)	28 (46.7)	17 (53.1)	9 (47.4)	53 (46.1)	26 (47.3)	
≥100 000	165 (49.1)	26 (47.3)	31 (51.7)	15 (46.9)	10 (52.6)	57 (49.6)	26 (47.3)	
Maternal education college or postgraduate	303 (78.9)	46 (73.0)	55 (87.3)	29 (87.9)	17 (85.0)	108 (77.1)	48 (75.0)	.70
Maternal BMI, mean (SD)^f^	26.5 (5.9)	29.9 (6.8)	25.6 (5.5)	26.3 (5.5)	25.4 (6.8)	26.1 (5.6)	25.6 (5.3)	<.001
Child age, mean (SD), mo	6.6 (5.4)	7.7 (6.9)	5.0 (3.5)	8.5 (5.8)	6.8 (6.0)	6.6 (5.3)	6.2 (5.1)	.12
Maternal age, mean (SD), y	33.6 (4.4)	33.4 (4.3)	33.1 (4.1)	34.3 (3.0)	33.9 (4.0)	34.0 (4.7)	32.8 (5.0)	.01
Feedings per day, mean (SD)	7.7 (3.2)	7.8 (2.9)	7.7 (2.8)	7.0 (3.6)	7.7 (3.3)	8.4 (3.5)	6.6 (2.8)	.08
Data available	370 (96.4)	62 (98.4)	57 (90.5)	33 (100)	20 (100)	136 (96.5)	62 (96.9)	NA

Abbreviations: ADs, anti-inflammatory drugs; BMI, body mass index; MABs, monoclonal antibodies; NA, not applicable; SSRIs, selective serotonin reuptake inhibitors.

^a^ Variables without information on data availability had no missing data.

^b^ Composed of a total of 67 in the disease-matched control group with only inflammatory disorders, 28 with only mood disorders, and 46 with both.

^c^ Calculated with χ^2^ and Kruskall-Wallis test for differences between groups.

^d^ Includes American Indian and Native Hawaiian/Pacific Islander.

^e^ A diagnosis of a psychiatric disorder or Edinburgh Postnatal Depression Scale (EPDS) score of 10 or more within 60 days from milk sampling.

^f^ Calculated as weight in kilograms divided by height in meters squared.

### Statistical Analysis

First, the distributions of the separate macronutrients (carbohydrates, protein, fat, and total energy) in the entire cohort of 424 analyzed samples were analyzed, and 40 samples were excluded due to outliers outside of ±2 SD from the mean in 1 or more macronutrient levels. The crude levels of macronutrients were compared between groups with independent samples *t* tests, and the background characteristics were compared between groups using Fisher exact test and Pearson χ^2^, as appropriate. After exclusion of outliers, the distributions of macronutrient levels were found to be approximately normal across the exposure groups, with approximately homogenous variances. For carbohydrates, the tails were somewhat more extended. However, considering the very similar mean carbohydrate levels across all exposure groups we estimated, excluding further cases with more modest deviating levels less than 2 SDs would not change the results. Therefore, the adjusted means and their 95% CIs for macronutrient levels between exposed vs healthy and exposed vs DMCs were compared in separate 1-way analyses of covariance for each exposure group. The analyses were adjusted for infant and maternal age, parity, maternal BMI, infant sex, exclusive breastfeeding, times fed per day, collection time of milk sample, maternal use of cannabis, and maternal occupation. The level of statistical significance was set as a 2-sided *P* < .05. Cases with missing data on included covariates were excluded from the adjusted analyses. Statistical analyses were performed with SPSS Statistics for Windows, Version 29.0 (IBM Corp). Data were analyzed from March to June 2024.

## Results

The final sample consisted of 384 milk samples, with 63 exposed to SSRIs, 63 to MABs, 33 to systemic steroids, and 20 to other anti-inflammatory drugs; 141 were from DMCs and 64 healthy unmedicated mothers (Figure 1). Maternal, infant, breastfeeding, and sample collection characteristics are presented in Table 1. A total of 194 infants (50.5%) were female, and 27 of the mothers (7.0%) were Asian, 48 (12.5%) non-Hispanic Black, 48 (12.5%) Hispanic, 291 (75.8%) non-Hispanic White, and 11 (2.9%) other. The mean (SD) maternal age in the entire cohort was 33.5 (4.4) years and infant age was 6.6 (5.4) months, with no significant difference between exposure groups on infant age (*F*_7_ = 11.60; *P* = .12), but with healthy control mothers being 0.3 to 1.5 years younger than the exposed and the DMC mothers (*F*_7_ = 17.63; *P* = .01).

Mothers treated with SSRIs had significantly higher mean (SD) BMI than mothers in the other groups (29.9 [6.8] compared with 25.6 [5.5] in mothers treated with MABs, 26.3 [5.5] with steroids, 25.4 [6.8] with other ADs, 26.1 [5.6] in DMC mothers, and 25.6 [5.3] in healthy mothers; *F*_7_ = 30.05, *P* < .001). The frequencies of cannabis use were lower among mothers treated with anti-inflammatory drugs than among the other groups (0 [0.0%], 2 [3.2%], and 2 [10.0%] among mothers treated with steroids, MABs, and other ADs, respectively, compared with 14 [22.2%], 24 [17.0%], and 17 [26.6%] among mothers treated with SSRIs, DMC mothers, and healthy mothers, respectively; *F*_7_ = 73.7; *P* < .001). No statistically significant differences were found between groups in race and ethnicity, parity, infant sex, exclusive breastfeeding, milk collection time, use of tobacco, and the measured socioeconomic factors (Table 1). Data on sample collection time were missing for 45 (11.7%), on maternal occupation for 38 (9.9%), on household income for 48 (12.5%), and on number of feeds per day for 14 (3.6%) of the participants.

**Figure 2.**
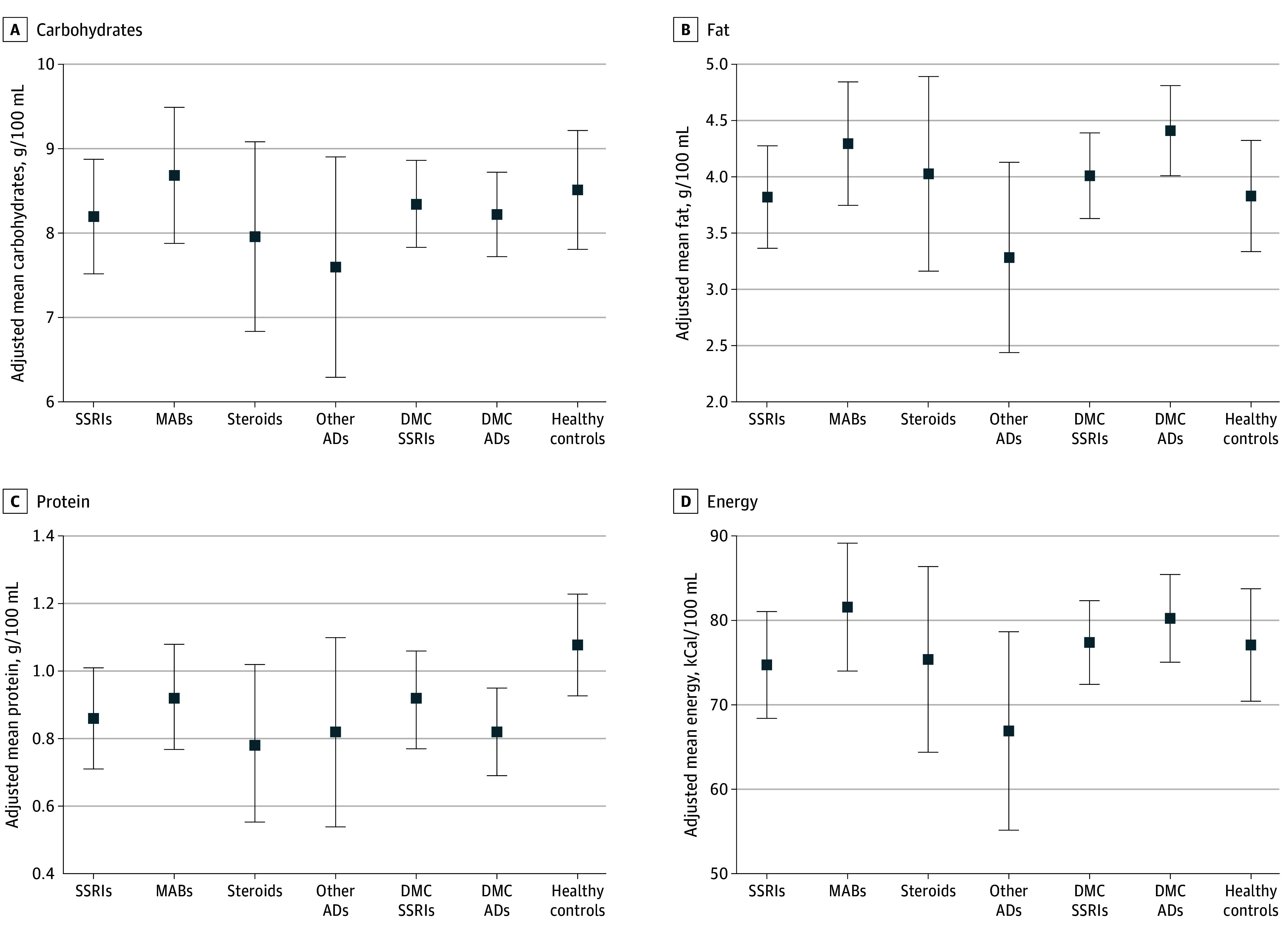
Adjusted Mean Content of Macronutrients in Human Milk Adjusted mean contents of A, carbohydrates; B, fat; C, protein measured in g/100 mL; and D, total energy measured in kcal/100 mL among the 63 mothers treated with SSRIs, the 63 treated with MABs, the 33 treated with systemic steroids, the 20 treated with ADs, the 73 DMC SSRIs, the 113 DMC ADs, and the 64 in the healthy control group. Error bars indicate 95% CIs. ADs indicates other anti-inflammatory drugs; DMC, disease-matched control group; MABs, monoclonal antibodies; SSRIs, selective serotonin reuptake inhibitors.

The proportion of participants with mood disorders was lower among mothers treated with SSRIs than among the DMCs they were compared with (58 of 63 [92.1%] vs 74 of 74 [100%]; *F*_1_ = 6.10; *P* = .02), and the proportions of participants with inflammatory disorders were lower in all AD exposed groups than in the DMCs they were compared with (proportion among DMCs: 112 of 113 [99.1%], MABs: 58 of 63 [92.1%]; *F*_1_ = 6.11; *P* = .02 compared with DMCs; systemic steroids: 15 of 33 [45.5%]; *F*_1_ = 64.97; *P* < .001 compared with DMCs; and other ADs 18 of 20 [90.0%]; *F*_1_ = 6.40; *P* = .06 compared with DMCs). The mean (SD) levels in the final cohort were 8.41 (2.19) g/100 mL for carbohydrates, 4.06 (1.74) g/100 mL for fat, 0.92 (0.53) g/100 mL for protein, and 78.15 (22.35) kcal/100 mL for total energy. The crude mean levels of carbohydrates ranged between 7.9 and 8.5 g/100 mL between the groups, and neither the crude nor the adjusted levels differed significantly between the groups (Table 2, Figure 2A). The crude mean protein content was 15% to 21% lower in samples from all exposed groups compared with those from healthy mothers (mean [SD] SSRIs, 0.92 [0.56]; MABs, 0.85 [0.51]; steroids, 0.88 [0.37]; other ADs, 0.85 [0.54] vs healthy mothers, 1.08 [0.50] g/100 mL), but after adjustments, the difference was only significant for SSRIs and steroids (*F*_1, 91_ = 4.32; *F*_1, 59_ = 5.00; *P* = .04 and *P* = .03, respectively) (Table 2, Figure 2C).

**Table 2.** Crude and Adjusted Mean Levels of Macronutrients in Milk Samples From Mothers Treated With Selective Serotonin Reuptake Inhibitor Antidepressants and Anti-Inflammatory Drugs, as Well as Untreated Disease-Matched Control Group (DMC) and Healthy Control Group

Exposure group	No.	Mean (SD)	Adjusted mean (95% CI)^a^	*P* value			
				Exposed vs controls		Exposed vs DMC	
				Crude	Adjusted	Crude	Adjusted
Carbohydrates, g/100 mL							
SSRIs	63	8.37 (2.13)	8.20 (7.52-8.88)	.51	.43	.92	.93
MABs	63	8.77 (2.42)	8.68 (7.88-9.49)	.82	.63	.26	.70
Steroids	33	7.89 (1.30)	7.96 (6.84-9.08)	.20	.41	.17	.89
Other ADs	20	7.93 (1.57)	7.60 (6.29-8.90)	.35	.20	.47	.44
DMC SSRIs	74	8.41 (1.92)	8.34 (7.83-8.86)	NA	NA	NA	NA
DMC ADs	113	8.34 (2.42)	8.22 (7.72-8.72)	NA	NA	NA	NA
Healthy control group	64	8.48 (2.44)	8.51 (7.81-9.22)^b^	NA	NA	NA	NA
Fat, g/100 mL							
SSRIs	63	3.96 (1.74)	3.82 (3.37-4.28)	.17	.95	.60	.66
MABs	63	4.26 (1.70)	4.30 (3.75-4.84)	.04	.16	.69	.55
Steroids	33	3.93 (1.89)	4.03 (3.16-4.89)	.84	.74	.24	.36
Other ADs	20	3.40 (1.21)	3.28 (2.44-4.13)	.26	.27	.03	.01
DMC SSRIs	74	4.11 (1.48)	4.01 (3.63-4.39)	NA	NA	NA	NA
DMC ADs	113	4.38 (1.90)	4.41 (4.01-4.81)	NA	NA	NA	NA
Healthy control group	64	3.85 (1.66)	3.83 (3.34-4.32)^b^	NA	NA	NA	NA
Protein, g/100 mL							
SSRIs	63	0.92 (0.56)	0.86 (0.71-1.01)	.09	.04	.89	.81
MABs	63	0.85 (0.51)	0.92 (0.77-1.08)	.01	.35	.95	.30
Steroids	33	0.88 (0.37)	0.78 (0.55-1.02)	.05	.03	.74	.80
Other ADs	20	0.85 (0.54)	0.82 (0.54-1.1)	.09	.12	.98	.78
DMC SSRIs	74	0.93 (0.54)	0.92 (0.77-1.06)	NA	NA	NA	NA
DMC ADs	113	0.85 (0.59)	0.82 (0.69-0.95)	NA	NA	NA	NA
Healthy control group	64	1.08 (0.50)	1.08 (0.93-1.23)^b^	NA	NA	NA	NA
Energy, kcal/100 mL							
SSRIs	63	77.04 (23.32)	74.73 (68.40-81.05)	.51	.43	.92	.93
MABs	63	81.30 (23.40)	81.57 (74.00-89.14)	.82	.63	.26	.70
Steroids	33	74.57 (19.33)	75.38 (64.37-86.38)	.20	.41	.17	.89
Other ADs	20	69.56 (15.34)	66.90 (55.15-78.66)	.35	.20	.47	.44
DMC SSRIs	74	78.64 (17.45)	77.38 (72.42-82.33)	NA	NA	NA	NA
DMC ADs	113	80.60 (24.70)	80.24 (75.04-85.44)	NA	NA	NA	NA
Healthy control group	64	77.16 (22.08)	77.08 (70.43-83.74)^b^	NA	NA	NA	NA

Abbreviations: ADs, anti-inflammatory drugs; MABs, monoclonal antibodies; NA, not applicable; SSRIs, selective serotonin reuptake inhibitors.

^a^ Calculated with 1-way analysis of covariance separate for each exposure group compared with the disease-matched control group for the medication and to the healthy control group, adjusted for infant and maternal age, parity, maternal body mass index, infant sex, exclusive breastfeeding, times fed per day, collection time of milk sample, maternal use of cannabis, and maternal occupation.

^b^ Adjusted means for the healthy control group are the means of the adjusted means for the healthy control group from all the pairwise comparisons between the exposure groups and healthy control group.

The crude mean fat and total energy were 10% to 22% lower in samples from mothers treated with other ADs (mean [SD] fat, 3.40 [1.21] g/100 mL and energy, 69.56 [15.35] kcal/100 mL) than in samples from the DMCs (4.38 [1.90] g/100 mL and 80.60 [24.70] kcal/100 mL) and healthy control group (3.85 [1.66] g/100 mL and 77.16 [22.08] kcal/100 mL) (Table 2, Figure 2B and D). However, after adjustments for the previously mentioned covariates, the difference between groups was only significant for the levels of fat and only when compared with the DMC (*F*_1, 88_ = 6.22; *P* = .01).

## Discussion

To our knowledge, this study was the first to examine associations between long-term maternal medication use and the macronutrient composition of human milk. We found that all macronutrients, apart from carbohydrates, differed to some degree in samples of human milk from mothers exposed to medications compared with the unexposed mothers. All mean values were within the reference range, but individual samples across the cohort had concerningly low levels of the different macronutrients.

Little is known about the outcomes of different exposures on the macronutrient composition of human milk. Altered dietary fat intake is known to change the composition of fatty acids in human milk, but the correlation between diet and levels of the other macronutrients is not clarified, and therefore, the clinical implications of this for the breastfed infant are so far unclear.^17,27^ There are also different aspects of maternal nutrition apart from the current dietary intake that could impact the composition of human milk, including nutrient stores and the ability to efficiently use nutrients, which may be influenced by hormonal changes during lactation.^17^ Therefore, it is possible that factors such as maternal diet and disease severity, potentially through maternal diet, could also affect the composition of macronutrients in human milk. If this is the case, these factors may represent unmeasured confounders that were not addressed in this analysis.

Whether the 20% differences in mean levels of protein and fat that were found are clinically significant and could impose health risks for the breastfed infants is not clear. It is likely that healthy infants would compensate for these lower levels of nutrients in human milk with intensified feeding, leading to increased milk production.^28^ However, for exclusively breastfed preterm and ill infants, these differences could potentially be sufficient to affect infant growth, and if not caught by routine monitoring of the infant, their health.

### Strengths and Limitations

This study had several strengths. A prospective cohort like this provides the possibility to collect in-depth data on important variables such as detailed information on maternal exposures, occupation level, and breastfeeding practices. Important variables related to the collection of the milk sample, such as time of day, are also thoroughly documented, allowing for more correct adjustments of the results. The sample sizes of mothers exposed to SSRIs and MABs were also estimated to be sufficient for finding larger differences in macronutrient levels.

This study also had limitations. The limited sample size regarding exposures to steroids and other anti-inflammatory drugs limited our interpretation of the results from these analyses. Another potential limitation is that the narrow definition of the exposure time window of 14 days before sample collection might have led to some misclassification of the exposed, although all were considered chronic users during the 2-week time window. In addition to this, mothers treated with MABs taken every 4 to 8 weeks were considered as exposed during this time period even if they had not taken the medication in the 14-day period if they were taking the medication regularly and were in steady state at the time of the milk sample collection.

There is also some uncertainty regarding the accuracy of the near-infrared milk analyzer used in our study.^12^ This should not have caused bias in any specific direction as inaccuracies would be expected to be nondifferential, but it may have attenuated the results. The accuracy of these easy-access milk analyzers needs to be improved further, both to increase the reliability of scientific studies on the effects of external exposures, and for clinical needs when expressed milk is fortified based on macronutrient analyses.^11^

## Conclusions

Maternal treatment with the studied medications was associated with lower mean levels of some macronutrients in human milk. Treatments with SSRIs, MABs, and systemic steroids were associated with significantly lower mean levels of protein, but for MABs, the difference was no longer significant after adjustment for clinically relevant covariates. Milk samples from mothers treated with other ADs had significantly lower mean levels of fat than samples from DMCs. Low nutritional levels in human milk could negatively affect infant growth and impose health risks for the breastfed infant. However, the causality of these findings cannot be determined before the effects of other maternal exposures, such as maternal diet and the severity of the underlying disorder, are clarified. We conclude that as the mean macronutrient levels were within normal range in all exposure groups, these results should not affect the breastfeeding recommendations for mothers treated with antidepressants and anti-inflammatory medications, and the growth of their breastfed infants should be monitored as per clinical routine.

## Notes

Supplement.
Data Sharing Statement
