# Supplementary material for: Macronutrients in Human Milk Exposed to Antidepressant and Anti-Inflammatory Medications
Source: JAMA Netw Open. 2025 Jan 7;8(1):e2453332. doi: 10.1001/jamanetworkopen.2024.53332 (PMC11707630; doi:10.1001/jamanetworkopen.2024.53332)
Supplement: Supplement. — Data Sharing Statement [file jamanetwopen-e2453332-s001.pdf]

Data Sharing Statement

Whaites Heinonen. Macronutrients in Human Milk Exposed to Chronic Medications. *JAMA Netw Open*. Published January 07, 2025. doi:10.1001/jamanetworkopen.2024.53332

Data
Data available: Yes
Data types: Other (please specify)
Additional Information: Requests for data should be submitted to the senior author (dr Chambers) and will be addressed as consistent with the underlying consent for use of data.
How to access data: chchambers@health.ucsd.edu
When available: With publication

Supporting Documents
Document types: None

Additional Information
Who can access the data: anyone requesting the data, per discretion of the senior researcher.
Types of analyses: per discretion of the senior researcher.
Mechanisms of data availability: per discretion of the senior researcher.
